# Open-Label Phase 1/2 Study of Daratumumab-Based Desensitization Before Kidney Transplantation

**DOI:** 10.1016/j.ekir.2024.08.020

**Published:** 2024-08-26

**Authors:** Caroline Pilon, Nizar Joher, Cédric Usureau, Emmanuelle Boutin, Anna Boueilh, Jean-Luc Taupin, Allan Thiolat, José L. Cohen, Vissal David Kheav, Florence Canoui-Poitrine, Maryvonnick Carmagnat, Philippe Grimbert, Marie Matignon

**Affiliations:** 1 Universite Paris Est Creteil, INSERM IMRB U955, Créteil, France; 2 Assistance Publique-Hôpitaux de Paris, Groupe hospitalo-universitaire Chenevier Mondor, Centre d’Investigation Clinique Biotherapy, Fédération hospitalo-Universitaire, Innovative therapy for immune disorders, Créteil, France; 3 Department of Nephrology and Renal Transplantation, Assistance Publique-Hôpitaux de Paris, Groupe hospitalo-universitaire Chenevier Mondor, Fédération Hospitalo-Universitaire, Innovative therapy for immune disorders, Créteil, France; 4 Laboratoire d'Immunologie et Histocompatibilité, Hôpital Saint Louis, Paris, France; 5 INSERM UMR976, Institut de Recherche Saint-Louis, Université de Paris-Cité, Paris, France; 6 Assistance Publique-Hôpitaux de Paris, Hôpitaux Universitaires Henri Mondor-Albert Chenevier, Public Health Department and URC, Créteil, France

**Keywords:** lymphocytes, pharmacokinetics, translational, transplantation

## Abstract

**Introduction:**

The safety and benefit of the anti-CD38 monoclonal antibody daratumumab, which induces lysis of antibody-producing plasma cells in sensitized patients prior to kidney transplantation, remain to be determined.

**Methods:**

A 2-phase (1 and 2), monocentric open-label study was conducted to evaluate the month 6 (M6) safety and efficacy of daratumumab in kidney transplant candidates with calculated panel reactive antibody (cPRA) > 95%. In the first (safety) phase, we used 4-weekly escalating doses of daratumumab. Phase 2 tested desensitization with 8 weekly infusions of 16 mg/kg daratumumab. cPRA 10,000 was calculated considering only human leukocyte antigen (HLA) antibodies with mean fluorescence intensity (MFI) of > 10,000.

**Results:**

Nine patients were enrolled in phase 1 and 14 in phase 2. Safety analysis showed 4 serious non-treatment-emergent adverse events (non-TEAEs), 36 mild TEAEs, mostly infusion-related reactions, grade 1 and 2 (causing 2 temporary drug discontinuations), but no serious TEAEs. Significant reductions in anti-HLA antibodies were observed at month 3 (M3), with cPRA 10,000 (*P* = 0.003), number of anti-HLA (*P* < 0.001), maximum MFI (MFI max) (*P* = 0.053), and the sum of MFI (MFI sum) (*P* < 0.001), with complete return to baseline levels at month 12 (M12). At M6, 46.15% (19.22%–74.87%) and 76.92% (46.19%–94.96%) of patients showed sustained response (1% decrease in cPRA) for cPRA 2000 and 10,000, respectively. At month 1 (M1), immune cells (T-reg, CD8 + TEMRA, CD19 + CD138 + B cells, and NK cells) significantly decreased. At M3, other antibodies decreased significantly, but returned to baseline levels at M12, except for gamma globulins, without any infectious complications.

**Conclusion:**

The first use of daratumumab in desensitization demonstrated infusion-related adverse (AEs) events and rapid, albeit transient, reductions in anti-HLA antibodies, with less than 40% of durable responders, limiting its potential clinical use.

## Graphical abstract

**Figure:**
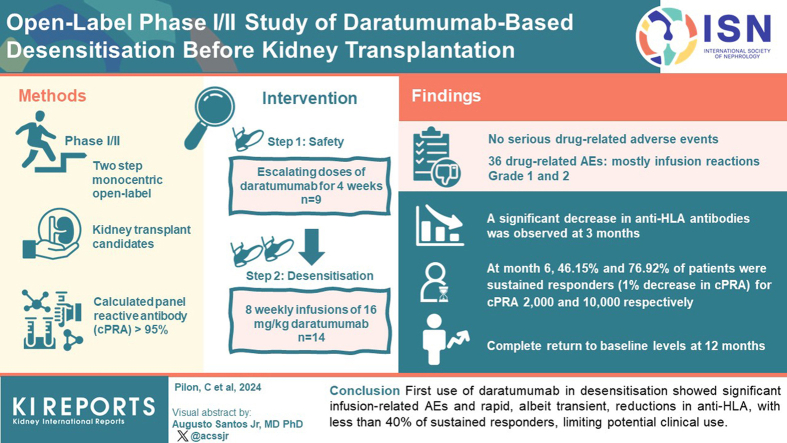

Although kidney transplantation is the best therapeutic option for end-stage renal disease, anti-HLA sensitization remains a major limitation to allograft access.^1^, ^2^, ^3^ Sensitization ranges from 0% to 99% as determined by the cPRA formula, which estimates the proportion of donors a tested candidate may be incompatible with.^1^^,^^2^ The higher the cPRA, the longer the time on the transplantation waiting list and the higher the risk of death before transplantation.^4^

Several strategies have been developed to improve access to transplantation for highly immunized kidney transplant candidates. For instance, prioritization according to cPRA threshold (85% in France vs. 98% in the US) has led to a significant increase in transplantation.^5^ However, the extremely sensitized individuals (cPRA > 99.9%) remain on the waiting list longer than the nonsensitized patients; thus, their higher mortality and morbidity.^4^^,^^6^ Another strategy is to use pleiotropic therapies to reduce antibody levels, such as high-dose i.v. Ig, rituximab, plasmapheresis, proteasome inhibitors, and imlifidase,^7^^,^^8^ which consequently increased the transplantation rates. Nevertheless, these therapies did not work for all patients, and in some cases the incidence of acute rejection after transplantation reached 40% with no significant survival benefit after desensitization as confirmed by a British study, the only European report in this concern.^7^^,^^9^ Therefore, there is an urgent need for additional and more effective therapies for patients with high cPRA.

Daratumumab, a human IgG1 monoclonal antibody that specifically binds to a unique epitope present on the CD38 molecule, induces, at least, a partial response in up to 30% of refractory multiple myeloma, due to its action on CD38-positive plasma cells, with a good safety profile.^10^ Plasma cells are the main cells responsible for the production of anti-HLA antibodies, and anti-CD38 antibodies have recently been used to inhibit the production of autoantibodies or alloantibodies.^11^, ^12^, ^13^ Kwun *et al.*^12^ previously demonstrated that daratumumab significantly reduces the secretion of anti-HLA antibodies in presensitized primates as well as in 2 patients, 1 with refractory acute kidney and heart antibody-mediated rejection (ABMR) and 1 awaiting heart transplantation refractory to conventional desensitization treatment.^12^ Since then, several case reports have confirmed their initial observations with successful desensitization, after which heart transplantation and effective treatment of refractory acute ABMR were possible.^14^, ^15^, ^16^, ^17^, ^18^

Very recently, another anti-CD38 antibody, isatuximab, has been used to desensitize patients awaiting kidney allograft and resulted in minimal decrease in cPRA despite a significant decrease in anti-HLA antibodies, in CD38+ plasmablasts, plasma cells, NK cells, and in HLA-specific IgG-producing memory B cells.^19^

Because there are currently no data on the use of daratumumab in highly sensitized patients awaiting kidney transplantation, we conducted a phase 1/2 study in which daratumumab was used for the first time in a cohort of such patients. The primary end point combined the safety of increasing doses of daratumumab and intrapatient cPRA variation by comparing cPRA levels at 6 months of treatment with its baseline levels.

## Methods

### Patients-Inclusion Criteria

We conducted a monocentric, open-label, phase 1/2 study on adult patients (>18 years) who had been waiting for a kidney transplant for at least 3 years and had a cPRA > 95%, in Henri Mondor hospital from October 2019 to June 2022. We selected patients whose cPRA was stable over the past 3 years to use each patient as their own control (mean SD was ± 2.4%). Exclusion criteria were contraindications to daratumumab treatment and/or renal transplantation. Based on the findings of Kwun *et al.*^12^ where daratumumab treatment can induce an increase in CD8 T-cells and promote T-cell mediated rejection and transient desensitization with anti-HLA rebound in animals, kidney transplantation was not allowed within the first 6 months of treatment.

All patients provided signed informed consent prior to enrollment. This study was approved by the hospital ethics committee and posted on the Clinical Trials website (NCT04204980). The study was an investigator-initiated study (MM, Principal Investigator), that is, designed, conducted, and analyzed solely by the investigators, with the approval of Janssen. All women of childbearing age underwent a pregnancy test, and active pregnancy was an exclusion criterion.

### Description of the Study

**Figure 1:**
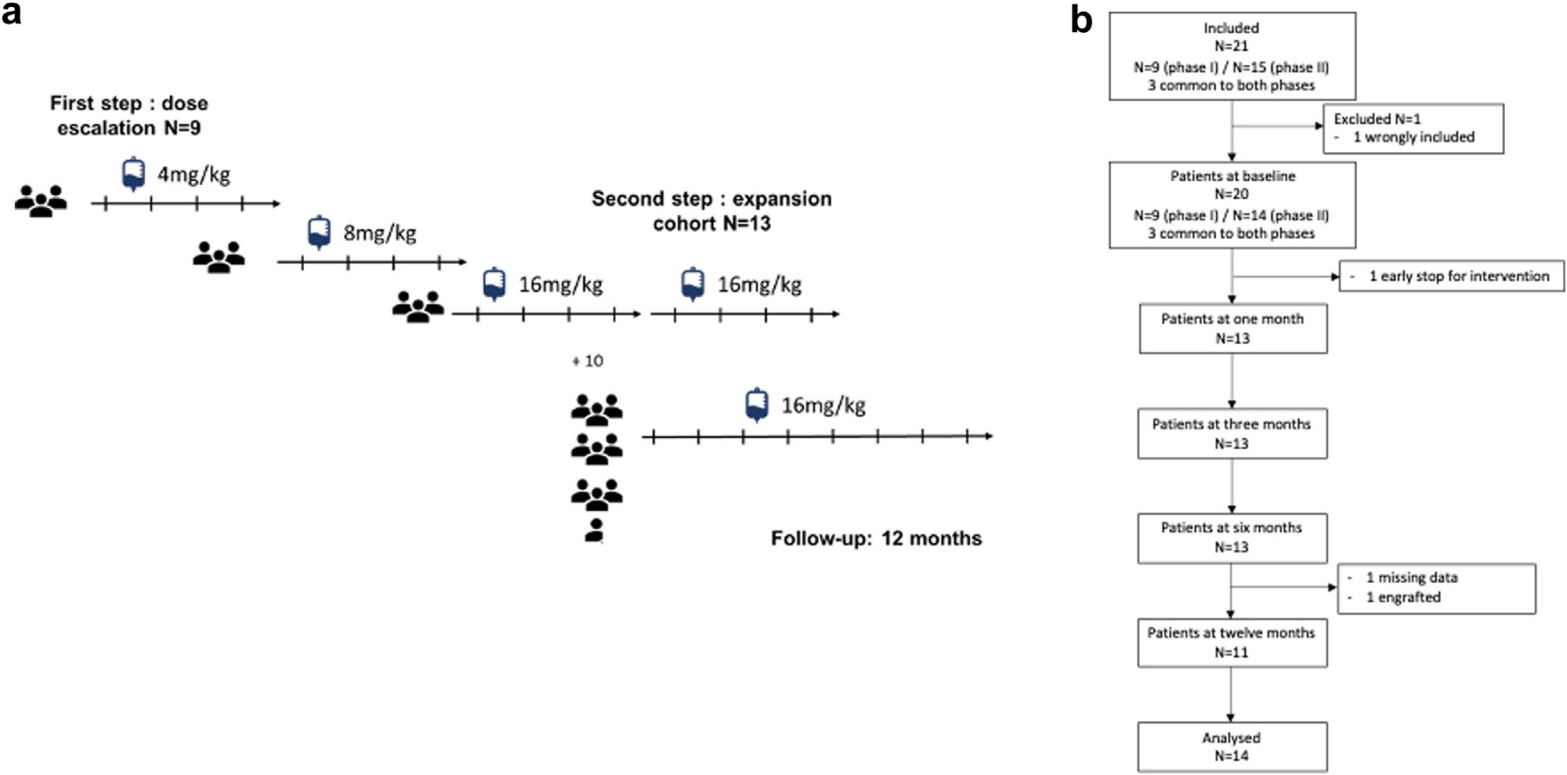
(a) Design and (b) flowchart of the study. The study consisted of 2 steps: phase 1 evaluated the safety of daratumumab given at doses that were doubled once every 4 weeks over 3 dose-intervals, from 4 mg/kg to 8 mg/kg and then to 16 mg/kg, in 9 patients (dose escalation phase). Phase 2 evaluated the desensitization effect of 16 mg/kg of daratumumab given for 8 weeks (desensitization step). Follow-up lasted 12 months after the first infusion.

The trial was designed to run in 2 steps. Phase 1 (dose escalation) evaluated the safety of daratumumab in patients who were given, for the first time in their lives, escalating doses of 4, 8, and 16 mg/kg/wk for 4 weeks per each dose, and step 2 (expansion) evaluated the desensitization effect of 16 mg/kg/wk of daratumumab given for 8 weeks (Figure 1a). The last 3 patients enrolled in phase 1 and treated with 4 weekly doses of 16 mg/kg daratumumab were enrolled as the first patients in phase 2 and had in total of 8 weekly infusions of 16 mg/kg daratumumab, given the good safety of the drug after the first 4 weekly infusions. Based on the hypothesis of a 10% reduction in intrapatient cPRA, a baseline cPRA of 95%, a SD of 0.1, and a 1-sided alpha risk of 5% for a power of 90%, we needed 13 patients in phase 2 to be followed for 12 months if no serious AEs (SAEs) occurred. Patients were replaced on a 1-to-1 basis in case of early withdrawal. Patients received only daratumumab in the study and specifically no other immunosuppressive agent or immunomodulatory molecule, plasma exchange, or immunoadsorption. The study included external monitoring. The follow-up lasted 12 months after the first infusion of daratumumab in all patients (phase 1 and 2), 11 months after the last dose of daratumumab in phase 1 patients and 10 months after the last dose of daratumumab in phase 2 patients.

### End Points

The primary end point of phase 1 was the incidence of SAEs, TEAEs, and non-TEAEs. The primary end point of phase 2 was intrapatient variation in cPRA measured M6 after finishing daratumumab treatment.

The secondary end points included AEs, SAEs, TEAEs, and non-TEAEs observed in phase 2; patient survival at M12 after enrolment, and intrapatient variation in cPRA at M3 and M12 of daratumumab treatment compared with baseline levels, percentage of sustained responders (had a decrease of at least 1% in cPRA at all time points); intrapatient variation in the sum of MFI (total, class 1, and class 2), MFI max (total, class 1, and class 2), and number of anti-HLA (total, class 1, and class 2) at M3, M6, and M12 of daratumumab treatment; percentage of patients transplanted between 6 and 12 months from enrollment; percentage of flow cytometry-measured blood immune cells at each assessment point; evolution of gamma globulins within the first year of daratumumab treatment (at M3, M6, and M12); evaluation of infectious events (invasive and opportunistic infections) at M6 and M12 from enrollment; evolution of vaccine-induced antibodies in patients with detectable antibody levels at enrollment, M3, M6, and M12 (anti-diphtheria and anti-pneumococcus); and evolution of intrapatient variation in ABO antibody titers at each assessment point compared with baseline.

We considered a 1% decrease in cPRA to be clinically significant because a decrease in cPRA from 99.9% to 99.8% or 99.7% could increase the transplantation rate by 12% and 20%, respectively.^20^ Our hospital laboratory calculated cPRA only as a round percentage figure without numbers after the decimal point.

### AEs and SAEs

The trial had a Data Safety and Monitoring Board that reviewed the safety of each patient in each interval of phase 1 before approving the next interval, and at the end of phase 1 before approving the expansion step. Dose-limiting toxicities were defined as AEs that would lead to treatment discontinuation, that is, infusion reactions that prevented resumption of the infusion regardless of the reaction duration; study TEAEs of the National Cancer Institute Common Terminology Criteria for Adverse Events grade 2 or higher triggered at the start of the infusion on any infusion day, except for grade 2 nonhematologic reactions that responded to symptomatic therapy and resolved within 6 hours of the its onset; study nonhematologic TEAEs of grade 3 or higher; grade 3 or higher hemolysis; study hematologic TEAEs of grade 4 or higher, except for grade 4 or higher platelet counts lasting less than 4 weeks; platelet counts of less than 50 × 10^9^/l occurring 4 weeks after the last infusion; platelet counts that decreased by 75% or more since visit 2 of phase 1 and remained > 25 × 10^9^/l but increased less than 10 × 10^9^/l above nadir 4 weeks after the last infusion. The Data Safety and Monitoring Board was consulted whenever SAEs occurred during the expansion phase.

### HLA Analyses

Sera collected before and after each treatment interval were analyzed for the presence of circulating anti-HLA antibodies directed against donor HLA-A, HLA-B, HLA-Cw, HLA-DR, or HLA-DQ antigens using high-resolution Luminex SAB assay technology (One Lambda, Inc., Canoga Park, CA) on a Luminex platform. All beads with a normalized MFI > 500 were considered positive. We used 2 cutoff points for MFI of anti-HLA antibodies to calculate PRA: 2000 and 10,000. Only anti-HLA antibodies with MFI > 2000 was included in the calculation of cPRA 2000, and only anti-HLA antibodies with MFI > 10,000 were included in the calculation of cPRA 10,000. The sum of MFI was defined as the sum of all MFI of all anti-HLA antibodies detected in the tested sera. The MFI max was the highest MFI of all anti-HLA antibodies in each serum. HLA data were standardized and normalized as recommended by the manufacturer (One Lambda); for normalization, negative control bead and serum were used. HLA data were centralized in a single laboratory in Paris (Saint Louis Hospital, Immunohistocompatibility). Global normalization further reduced MFI variation by almost 20% (30% for MFI between 500 and 1500 and 10% for MFI above 1500-personal data).

### Isohemagglutinin Analyses

Natural isohemagglutinins IgM and IgG anti-A/B were measured by flow cytometry. Red blood cells from blood group A and B donors were obtained from the French Blood Agency. Red blood cells were washed and resuspended in phosphate-buffered saline (PBS; Life Technologies; Thermo Fisher Scientific, Waltham, MA) to 10^5^ cells/μl and patient sera were treated with EDTA 0.01M (10 minutes at room temperature) for both IgG and IgM anti-A/B detection. Dithiothreitol reagent was used (5 mM at 37 °C for 20 minutes) for anti-IgG detection. PBS-suspended erythrocytes and treated sera were then incubated at 4 °C for 30 minutes. After 3 washes, 50 μl of FITC-conjugated F(ab')2 goat antihuman IgG or FITC-conjugated F(ab')2 goat antihuman IgM (Jackson Immunoresearch Labs) were added, and the solutions were incubated at 4 °C for 30 minutes. The red blood cells were washed 3 times with PBS and resuspended in 1% formaldehyde-containing PBS before acquisition of 10,000 events on a Becton Dickinson FACS Canto II flow cytometer. Results were expressed as the ratio of the MFI of the serum to the MFI of the negative control.

### Blood Immune Cell Phenotype

Peripheral blood was drawn from patients before the first daratumumab infusion and then at M1, M3, M6, and M12 after enrollment. Peripheral blood mononuclear cells (PBMCs) were isolated using lymphocyte separation medium (Eurobio, Les Ulis, France) and resuspended in PBS (Life Technologies; Thermo Fisher Scientific, Waltham, MA) containing 3% fetal bovine serum (FBS; Gibco, Life Technologies; Thermo Fisher Scientific). PBMCs were stained with different mAb combinations for 20 minutes at 4 °C in staining buffer (PBS with 3% FBS). The directly conjugated mAbs CD56-APC (clone B159), CD3-V450 (clone UCHT1), CD45RA-FITC (clone L48), CD27-V450 (clone M-T271), CD25-PE-Cy7 (clone 2A3), CD16-FITC (clone NKP15), CD38-PE (clone HB7), CD4-APC (clone RPA-T4), and CD8-V450 (clone RPA-T8) were provided by BD Biosciences (France); CD19-APC-Cy7 (clone HIB19) from Biolegend; CD138-PE (clone B-A38) and IgD-FITC (clone IADB6) from Beckman Coulter (France); and CCR7-PE-Vio770 (clone REA546) and CD38-PE-Vio770 (clone REA572) from Miltenyi Biotec. Staining with eFluor 450-labeled anti-Foxp3 (clone PCH101) was performed using the Foxp3 staining buffer eBioscience kit (Thermo Fisher Scientific) and protocol. Data were processed using FlowJo software (FlowJo LLC, Ashland, OR). The gating strategy is detailed in Supplementary Figure S1.

### Statistical Analysis

Statistical analysis followed CONSORT (Consolidated Standards of Reporting Trials) guidelines, and the efficacy and safety analyses were performed on an intention-to-treat basis for all cases. Phase 1 and phase 2 analyses were performed separately. Analyses of anti-HLA data were performed on an intention-to-treat basis with multiple imputation for patients with missing data, and on a per-protocol basis only for patients who were protocol compliant and treated. Demographic, clinical, and biological characteristics of patients were described using *N* (%) and median (Q1–Q3) for categorical and continuous variables, respectively.

Primary and secondary end points were reported as *N* (%) or median/mean ([Q1–Q3]/SD) with 95% confidence intervals, as appropriate.

In phase 2 sample, changes in cPRA, MFI max (total, class 1, and class 2), MFI sum (total, class 1, and class 2), and numbers of anti-HLA antibodies (total, class 1, and class 2) at M1, M3, M6, and M12 were compared with baseline levels using a mixed linear regression model with random intercepts for repeated measurements. Blood concentrations of antibodies, gamma globulins, vaccine-induced antibodies, and natural isohemagglutinins were described and compared using a mixed linear regression model with random intercepts for repeated measurements at M3, M6, and M12 (vs. baseline). All tests were 2-tailed and a *P*-value < 0.05 was considered significant. Data were analyzed with Stata SE STATA version 17.0 (StataCorp, College Station, TX).

## Results

### Study and patients

The study was run in 2 steps (Figure 1a). A flowchart of the patients enrolled in the study is shown in Figure 1b. In the dose escalation phase, the 9 patients enrolled were divided into 3 groups of 3 and treated with daratumumab at doses of 4, 8, and 16 mg/kg/wk for 4 weeks (phase 1). A total of 15 patients were enrolled in the expansion phase. Of these, 1 was incorrectly enrolled and did not receive any treatment (lung neoplasia diagnosed between baseline visit and first infusion), leaving 14 patients for the intention-to-treat analysis. The first 3 patients were from the last dose-interval of the first phase, had already been treated with 4 doses of 16 mg/kg/wk of daratumumab, and received 4 additional weekly doses of 16 mg/kg for a total of 8 weeks. One patient could not receive more than 1 infusion due to the development of an unspecified intestinal obstruction requiring surgery,^21^ that left 13 patients to complete the treatment (16 mg/kg/wk daratumumab for 8 weeks, phase 2). Demographic and anti-HLA characteristics at baseline is shown in Table 1. All patients had circulating class 1 and class 2 anti-HLA antibodies. All 14 patients (phase 2) completed the clinical follow-up at M12, and 1 patient, living in the French West Indies, did not have biological follow-up data (anti-HLA and non-anti-HLA antibodies, and PBMC analyses) at M12. For that patient, the last clinical data were collected by the local treating physician in the French West Indies, and through a telephone contact with the patient.

**Table 1:** Baseline demographic, clinical, and anti-HLA characteristics of participants

Participants	*n* = 9	*n* = 14

Sex, male, *n* (%)	6 (66.7)	8 (57.1)
Age (yr), median (IQR)	48.1 (44.1–51.4)	48.0 (41.0–59.1)
Initial nephropathy		
Glomerulopathy, *n* (%)	2 (22.2)	5 (35.7)
Diabetes	0 (0)	1 (7.1)
Vascular, *n* (%)	1 (11.1)	2 (14.3)
Interstitial, *n* (%)	0 (0)	0 (0)
Genetic, *n* (%)	0 (0)	1 (7.1)
Immunologic, *n* (%)	0 (0)	0 (0)
Others, *n* (%)	0 (0)	0 (0)
Undetermined, *n* (%)	6 (66.7)	5 (35.7)
Comorbidities		
Hypertension, *n* (%)	8 (88.9)	13 (92.9)
Diabetes mellitus, *n* (%)	2 (22.2)	3 (21.4)
Dialysis, *n* (%)	9 (100)	14 (100)
Duration, yr, median (IQR)	6.72 (4.38–7.24)	6.62 (3.82–9.75)
Kidney allograft waiting list		
Duration, yr, median (IQR)	6.06 (4.41–6.57)	5.48 (3.93–7.92)
Immunological risk		
Previous transplantation	7 (77.8)	13 (92.9)
Calculated panel reactive antibodies (cPRA)		
MFI 2000, %, median (IQR)	99 (97–100)	98 (85–100)
MFI 10,000, %, median (IQR)	96 ((83–100)	65 (56–84)
Anti-HLA antibodies		
Class 1	9 (100)	14 (100)
Class 2	9 (100)	14 (100)
Class 1 and class 2	9 (100)	14 (100)

cPRA, calculated panel reactive antibody; HLA, human leukocyte antigen; IQR, interquartile range; MFI, mean fluorescence intensity.

### SAEs, TEAEs, and Patient Survival

No TEAEs were observed in either phase (Table 2). Four non-TEAEs were observed: 1 death due to meningeal hemorrhage detected 5 months after enrollment, 1 unspecified bowel obstruction requiring surgery for which treatment was discontinued 1 week after the first infusion, 1 chronic left hemisphere subdural hematoma, and 1 pregnancy loss.

**Table 2:** Serious adverse events, treatment-emergent adverse events, and non-treatment-emergent adverse events

Patients, *n* (%)	Phase 1 *n* = 9	Phase 2 *n* = 14
Serious adverse events, *n* (%)	1 (7.7)	2 (14.3)
Time to event from first infusion, mo, median (IQR)	5	2.68 (0.46–4.90)
Time to event from last infusion, mo, median (IQR)	4.3	1.76 (0.23–3.29)
Related to treatment, *n* (%)	0 (0)	0 (0)
Not related to treatment, *n* (%)	1 (7.7)	2 (14.3)
Death because of meningeal hemorrhage, *n* (%)	1 (7.7)	0 (0)
Intestinal obstruction leading to surgery, *n* (%)	0 (0)	1 (50.0)
Miscarriage, *n* (%)	0 (0)	1 (50.0)
Adverse events, *n*	9	27
Patients, *n* (%)	5 (55.6)	9 (69.2)
Number per patient, median (IQR)	2 (2–2)	4 (2–4)
Time to event, days, median (IQR)	0 (0–0)	0 (0–2)
TEAEs, *n* (%)	7 (77.8)	22 (81.5)
Infusion-related reactions grade 1 or grade 2, *n* (%)	7 (100)	14 (63.6)
Nasal congestion, *n* (%)	0 (0)	1 (7.14)
Sore throat, *n* (%)	2 (28.57)	6 (42.86)
Allergic rhinitis, *n* (%)	0 (0)	1 (7.14)
Chest discomfort, *n* (%)	1 (14.29)	1 (7.14)
Pulmonary edema, *n* (%)	0 (0)	1 (7.14)
Bronchospasm, *n* (%)	2 (28.57)	3 (21.43)
Dyspnea, *n* (%)	1 (14.29)	1 (7.14)
Pruritus, *n* (%)	1 (14.29)	0 (0)
Leading to temporary infusion cessation, *n* (%)	0 (0)	2 (9.1)
Others, *n* (%)	0 (0)	6 (27.3)
Fatigue, *n* (%)	0 (0)	2 (33.3)
Sore throat, *n* (%)	0 (0)	2 (33.3)
Allergic rhinitis, *n* (%)	0 (0)	2 (33.3)
NTEAEs, *n* (%)	2 (22.2)	5 (18.5)
Others, *n* (%)	2 (100)	5 (100)
Blurred vision, *n* (%)	1 (50)	1 (20)
Stomach pain, *n* (%)	1 (50)	0 (0)
COVID-19, *n* (%)	0 (0)	1 (20)
Dyspnea, *n* (%)	0 (0)	1 (20)
Seizure, *n* (%)	0 (0)	1 (20)
Hyperkaliemia, *n* (%)	0 (0)	1 (20)

IQR, interquartile range; NTEAEs, nontreatment-emergent adverse events; TEAEs, treatment-emergent adverse events.

The most common cause of AEs was infusion-related reaction grade 1 or 2 with 7 (77.8%) and 14 cases (63.6%) in phase 1 and 2, respectively. The infusion had to be stopped in 2 patients but was resumed after 30 minutes and completed without further AEs. Patient survival at M12 was 94.8%.

### Anti-HLA Antibodies Characteristics

In Table 3, we show the anti-HLA characteristics of the 13 patients included in the desensitization phase. Because 1 patient was transplanted between M6 and M12 and 1 patient did not complete the biological follow-up at M12, only the data of the remaining 11 patients were included in the M12 analysis. Immune cell phenotype was analyzed in 10 patients (76.9%) at M6 and 8 patients (72.7%) at M12 (Table 4). Only anti-HLA antibodies with MFI > 2000 was included in the calculation of cPRA 2000 and only anti-HLA antibodies with MFI > 10,000 were included in the calculation of cPRA 10,000.

**Table 3:** Analysis of anti-HLA data for all cases (at baseline and follow-up time-points)

Variables	Baseline	Month 1	*P*-value (baseline-M1)	Month 3	*P*-value (baseline-M3)	Month 6	*P*-value (baseline-M6)	Month 12	*P*-value (baseline-M12)
Patients, *n*	13	13		13		13		11	
Blood sample, *n* (%)	13 (100)	13 (100)		13 (100)		13 (100)		11 (100)	
cPRA									
2000, %, median (IQR)	99 (95–100)	99 (92–99)	0.64	97 (88–99)	0.185	97 (93–99)	0.668	99 (93–100)	0.197
10,000, %, median (IQR)	74 (57–84)	68 (55–83)	0.059	67 (39–79)	0.003	62 (38–84)	0.074	79 (56–94)	0.399
MFI sum									
class 1 and class 2, median (IQR)	296654 (217711–459407)	302589 (194266–379182)	0.003	276503 (146348–317537)	<0.001	279677 (160798–314087)	0.005	318340 (168918–579956)	0.207
class 1, median (IQR)	224413 (148482–388411)	173032 (78688–316940)	0.006	127734 (67540–243921)	<0.001	156138 (80514–267207)	0.003	238425 (127409–471823)	0.262
class 2, median (IQR)	70996 (46681–131834)	61868 (34621–122022)	0.04	50571 (40731–117749)	0.009	48470 (45655–119766)	0.248	52793 (44828–147020)	0.307
MFI max									
class 1 and class 2, median (IQR)	21165 (20126–23940)	22532 (16733–23654)	0.336	21461 (14773–23277)	0.053	20887 (20524–22996)	0.17	22041 (20647–23414)	0.592
class 1, median (IQR)	19714 (16909–22267)	18832 (12252–21138)	0.143	16648 (12576–21392)	0.009	18975 (12063–20840)	0.036	19162 (15586–21092)	0.049
class 2, median (IQR)	20410 (14082–23116)	22041 (12407–23654)	0.543	21461 (8918–23277)	0.364	20869 (11033–21867)	0.78	22041 (8162–23414)	0.928
Anti-HLA number									
class 1 and class 2, median (IQR)	61 (45–81)	50 (35–64)	<0.001	45 (36–62)	<0.001	50 (34–67)	0.007	57 (48–72)	0.301
class 1, median (IQR)	43 (32–56)	37 (19–46)	<0.001	36 (21–47)	<0.001	37 (20–45)	0.003	39 (27–58)	0.533
class 2, median (IQR)	17 (7–29)	13 (11–18)	0.005	15 (6–21)	0.006	14 (12–22)	0.436	16 (14–25)	0.318

cPRA, calculated panel reactive antibody; HLA, human leukocyte antigen; IQR, interquartile range; M, month; MFI max, maximum mean fluorescence intensity; MFI sum, sum of mean fluorescence intensity.

**Table 4:** Evolution of immune cells during the study period

	Baseline	M1	*P*-value (baseline-M1)	M3	*P*-value (baseline-M3)	M6	*P*-value (baseline-M6)	M12	*P*-value (baseline-M12)
Patients, *n*	13	13		13		13		11	
Blood sample, *n* (%)	10 (76.9)	10 (76.9)		10 (76.9)		10 (76.9)		8 (72.7)	
Cells population									
T-cells (CD3+), % (IQR)	70.7 (54.7–76.7)	68.0 (55.4–80.5)	0.985	67.3 (55.4–80.4)	0.630	66.1 (57.9–78.8)	0.940	77.5 (55.0–81.9)	0.243
CD4+	61.2 (45.7–66.7)	61.5 (47.4–73.3)	0.463	59.6 (38.9–72.9)	0.882	58.5 (47.9–70.8)	0.961	60.1 (47.6–64.1)	0.072
Naives (CD45RA+ CCR7+)	43.2 (33.1–48.5)	49.3 (39.6–55.8)	0.068	33.5 (28.4–41.5)	0.0763	41.1 (29.1–44.8)	0.4157	40.4 (32.4–49.2)	0.6354
TEMRA (CD45RA+ CCR7−)	4.72 (4.36–5.50)	5.40 (3.61–8.22)	0.770	8.26 (4.17–13.3)	0.0273	6.39 (4.74–11.1)	0.0547	4.24 (2.56–9.32)	0.8438
Central memory (CD45RA− CCR7+)	21.7 (13.6–30.7)	21.5 (16.8–30.2)	0.607	20.4 (15.6–27.9)	0.9040	20.3 (11.2–35.3)	0.6871	17.3 (15.4–21.5)	0.4253
Effector memory (CD45RA− CCR7−)	32.0 (23.3–38.6)	22.1 (17.0–31.1)	0.037	31.6 (26.7–35.5)	>0.999	33.3 (27.2–42.0)	0.5347	34.6 (23.8–43.3)	0.3392
T-reg (CD4+ Foxp3+)	7.47 (6.00–10.4)	5.58 (1.69–9.29)	0.027	6.57 (5.40–7.69)	0.037	6.18 (3.89–8.22)	0.098	6.89 (6.02–11.1)	0.945
CD8+	12.1 (10.0–18.4)	16.7 (11.5–44.5)	0.120	16.7 (11.0–32.4)	0.0699	15.2 (8.92–25.3)	0.1786	14.3 (12.1–19.3)	0.270
Naives (CD45RA+ CCR7+)	25.2 (15.5–29.3)	47.6 (35.4–58.4)	0.001	31.3 (10.6–43.6)	0.3996	25.6 (3.14–34.2)	0.6306	16.5 (9.43–20.7)	0.1606
TEMRA (CD45RA+ CCR7−)	59.6 (53.1–64.5)	29.2 (27.1–43.5)	0.0003	45.9 (36.6–66.4)	0.3841	59.5 (49.6–75.2)	0.7070	70.2 (57.9–76.1)	0.0124
Central memory (CD45RA− CCR7+)	0.69 (0.08–1.03)	2.16 (1.93–3.65)	0.037	1.10 (0.46–2.27)	0.5566	0.41 (0.21–0.72)	0.3008	0.92 (0.43–1.79)	0.7422
Effector memory (CD45RA− CCR7−)	11.3 (8.59–22.8)	15.6 (10.3–21.5)	0.770	16.6 (7.29–20.7)	0.9219	10.2 (9.79–15.1)	0.6523	12.8 (10.1–17.3)	0.3125
B-cells (CD19+), % (IQR)	7.20 (2.50–9.64)	5.258 (2.31–9.27)	0.415	3.28 (2.11–5.76)	0.0099	6.88 (1.64–7.69)	0.0535	3.55 (2.90–7.23)	0.843
CD138	1.07 (0.62–2.08)	0.28 (0.23–0.36)	0.002	0.76 (0.48–1.72)	0.375	0.85 (0.09–1.29)	0.242	1.19 (0.48–1.88)	0.945
Naives (IgD+ CD27−)	61.2 (48.8–74.6)	67.2 (43.2–77.6)	0.904	66.8 (41.8–75.2)	0.492	60.6 (49.0–68.4)	0.253	61.9 (43.1–81.8)	0.921
Unswitched memory (IgD+ CD27+)	6.64 (2.69–9.80)	4.75 (3.25–9.90)	0.695	5.43 (3.78–11.2)	0.946	7.12 (3.05–12.8)	0.540	6.50 (2.25–10.8)	0.112
Switched memory (IgD− CD27+)	20.9 (13.2–29.6)	19.9 (11.5–35.5)	0.308	21.5 (33.8–37.9)	0.063	24.5 (12.9–33.6)	0.115	22.7 (10.0–38.3)	0.331
NK cells (total CD56+), % (IQR)	13.1 (10.3–16.4)	3.48 (2.03–5.45)	<0.0001	5.88 (4.65–9.88)	0.001	7.37 (3.56–10.5)	0.0063	12.1 (8.37–18.2)	0.7300
CD56+ CD16+	7.13 (5.36–10.9)	0.63 (0.48–0.77)	0.0003	0.82 (0.54–1.30)	0.0005	0.85 (0.15–1.68)	0.0039	4.38 (1.50–7.00)	0.143
CD56+ CD3−	9.66 (5.95–12.8)	1.43 (1.02–1.93)	0.0002	1.81 (1.61–2.51)	0.0005	2.19 (1.48–2.74)	0.0098	6.45 (3.43–8.05)	0.2105
NKT cells CD56+ CD3+	3.20 (1.90–4.63)	1.47 (1.06–4.63)	0.0059	4.27 (3.60–8.22)	0.0952	5.65 (2.56–11.9)	0.1701	5.74 (4.17–10.4)	0.0356

IQR, interquartile range; M, month.

**Figure 2:**
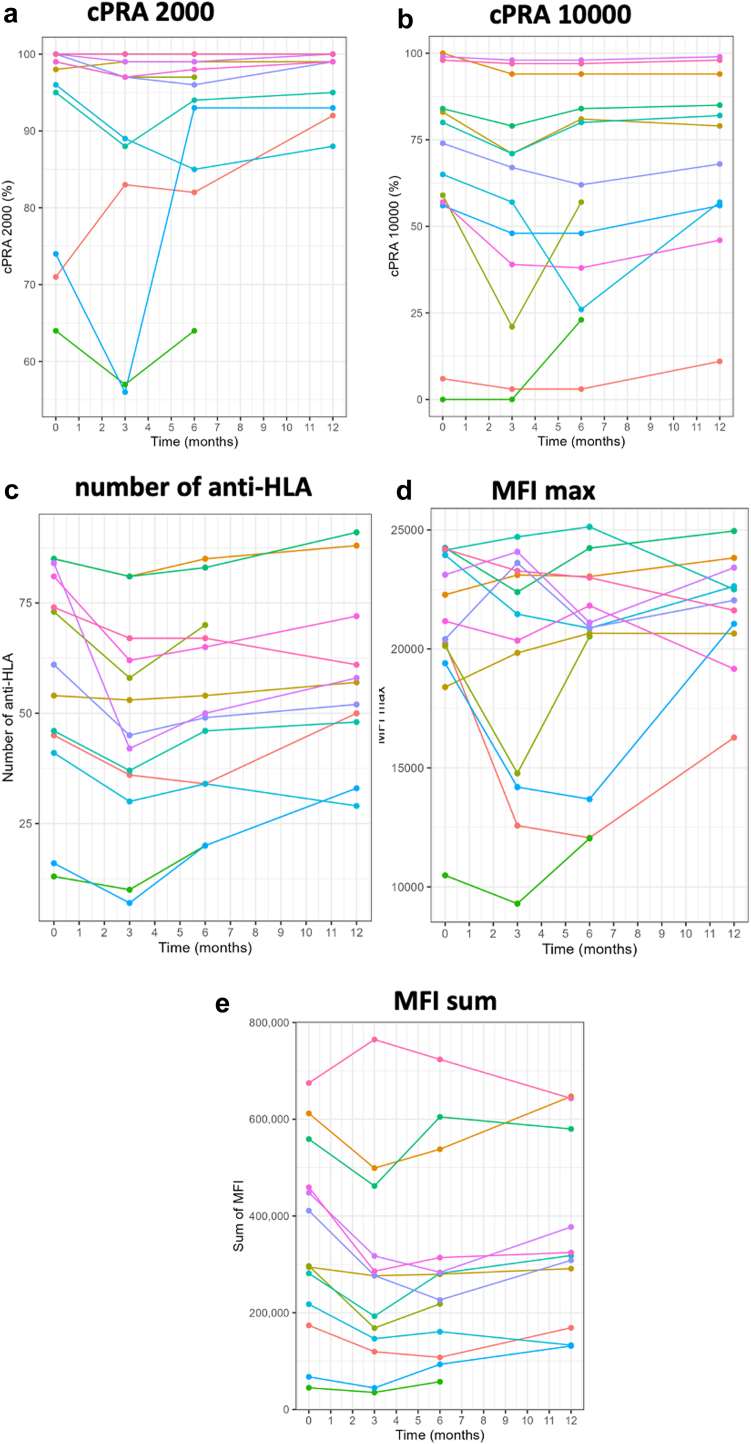
Evolution of anti-HLA characteristics between baseline and month 12 after the first infusion; each curve represents a patient. (a) cPRA 2000 remained stable (*P* = 0.197); (b) cPRA 10,000 returned to baseline (*P* = 0.399); (c) total number of anti-HLA antibodies returned to baseline (*P* = 0.301); (d) global MFI max returned to baseline (*P* = 0.592); (e) global MFI sum returned to baseline (*P* = 0.207). cPRA, calculated panel reactive antibody; MFI max, maximum mean fluorescence intensity; MFI sum, sum of mean fluorescence intensity.

At M3, almost all anti-HLA antibody characteristics significantly decreased compared with baseline, that is, cPRA 10,000 (*P* = 0.003), number of anti-HLA antibodies, total, class 1 or class 2 (*P* < 0.001, *P* < 0.001, and *P* = 0.006, respectively), total and class 1 MFI max (*P* = 0.053 and *P* = 0.009, respectively) and total, class 1 and class 2 MFI sum (*P* < 0.001, *P* < 0.001, and *P* = 0.009, respectively) (Supplementary Figure S2, Figure 2, and Table 3). In addition, for almost all the above anti-HLA parameters, this decrease started earlier, at 1 month after the first infusion. The results were similar in intention-to-treat analyses using multiple imputation and in per-protocol analyses (Supplementary Table S1).

At M6, the trend continued with significant decreases in total, class 1 MFI sum, class 1 MFI max, total and class 1 number of anti-HLA antibodies (*P* = 0.005, *P* = 0.003, *P* = 0.036, *P* = 0.007, and *P* = 0.003, respectively) (Supplementary Figure S3, Figure 2, and Table 3). Per-protocol analysis showed a similar decrease in cPRA 10,000 at M6 (Supplementary Table S1). At M12, all anti-HLA parameters returned to the levels observed at baseline, except for MFI max in class 1, which remained significantly lower than baseline (*P* = 0.049) (Figure 2 and Table 3). The same results were obtained in the per-protocol analysis where class 1 MFI max remained significantly lower (Supplementary Table S1).

For cPRA 2000, 46.15% (19.22%–74.87%) of patients (*n* = 6/13) could be qualified as sustained responders (had at least 1% reduction) at M6, and 18.18% (2.28%–51.78%) (*n* = 2/11) at M12. For cPRA 10,000, 76.92% (46.19%–94.96%) of patients (*n* = 10/13) could be qualified as sustained responders at M6, and 45.45% (16.75%–76.62%) (*n* = 5/11) at M12.

### Immune Cell Phenotype Changes During the Study Period

**Figure 3:**
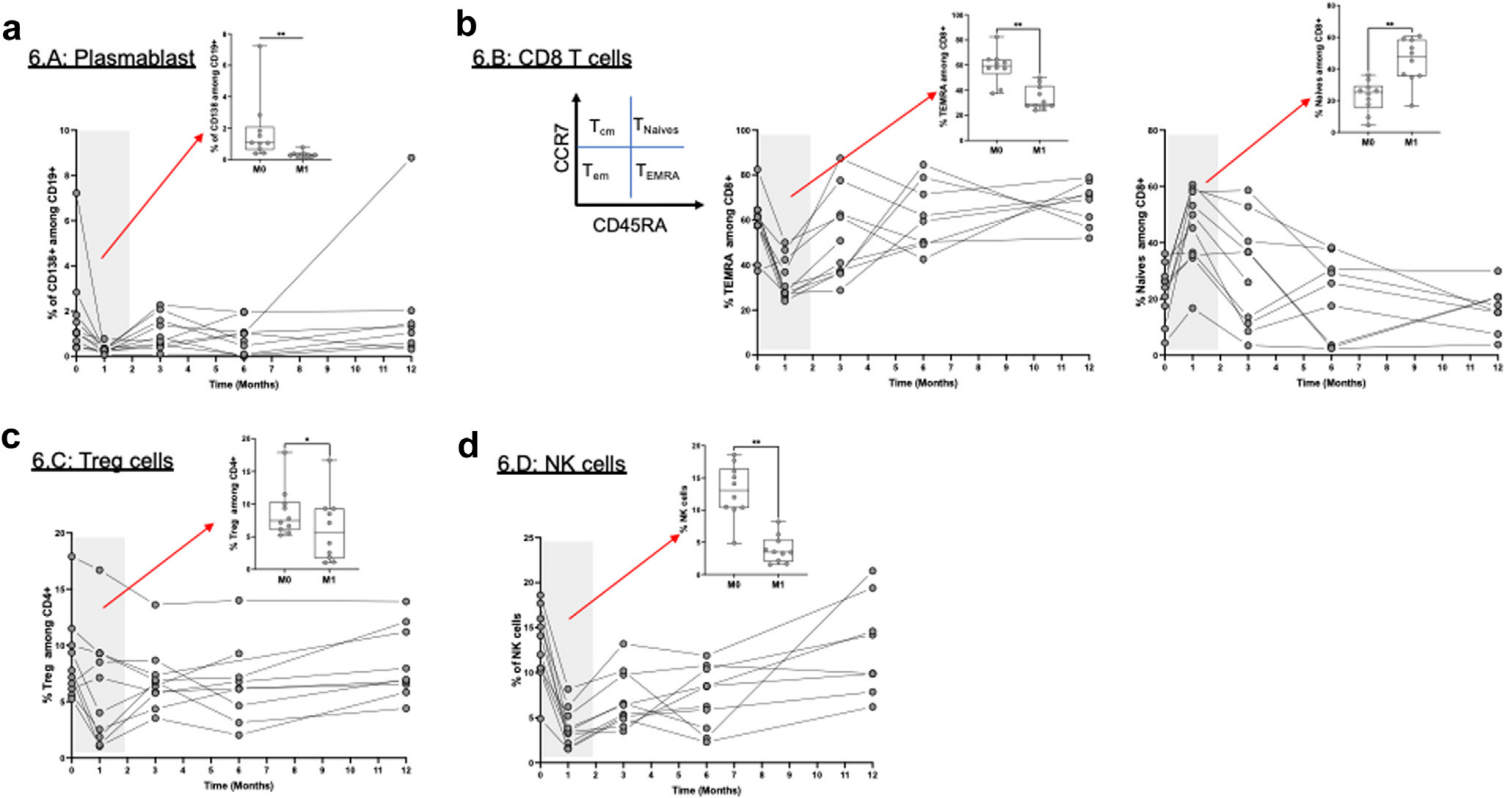
Phenotypic analysis of peripheral blood mononuclear cells (PBMCs), kinetics of the indicated population over the 12-month follow-up and box plot comparison for the M0/M1 period. (a) Plasmablast CD138+ cell levels among PBMC. (b) CD8 CD45RA+ CCR7− TEMRA (terminally differentiated effector memory) and CD45RA+ CCR7+ naive cell levels among CD8. (c) Percentage of T-reg cells (CD4+ CD25+ Foxp3+) among CD4 cells. (d) Total NK cells (CD56+) among PBMC. Box plots show the percentage of positive cells with 5% to 95% whiskers. Wilcoxon signed rank test was used to compare results. ∗*P* < 0.05, ∗∗*P* < 0.01. M0, month 0; M1, month 1; NK, natural killer; Treg, T-regulator cells.

In Figure 3 and Table 4, we show immune cell phenotypes at several time points during the 12-month study period, all compared with baseline levels. Only phase 2 patients (receiving 16 mg/kg) were included in this analysis. The first 3 patients did not have interpretable pre-daratumumab samples due to experimental issues; thus, we included the remaining 10 patients, who had pre daratumumab samples, in the final analysis. At M12, the end of follow-up period, only 8 patients had available samples; for the other 2 patients, 1 was transplanted and excluded because of possible immune cell changes induced by immunosuppressive treatment, and 1 did not complete biological follow-up at M12. At M1, the levels of T-cells CD4+ effector memory (CD45RA- CCR7-), T-reg (CD4+ Foxp3+), CD8+ terminally differentiated effector memory (TEMRA) (CD45RA+ CCR7−), B-cells CD19+ CD138+, and all types of NK cells significantly decreased, whereas T-cells CD8+ naive (CD45RA+ CCR7+), and CD8+ central memory (CD45RA− CCR7+) significantly increased. At M3, the levels of T-cells CD4+ TEMRA (CD45RA+ CCR7−) continued to increase significantly (*P* = 0.0273), and those of T-reg (CD4+ Foxp3+), B-cells CD19+, NK cells CD56+, NK cells CD56+CD16+, and NK cells CD56+ CD3− remained lower (*P* = 0.037, *P* = 0.009, *P* = 0.001, *P* = 0.0005, and *P* = 0.0005, respectively). At M6, the decrease in total NK cells CD56+, NK cells CD56+ CD16+, and NK cells CD56+ CD3− persisted (*P* = 0.0063, *P* = 0.0039, and *P* = 0.0098, respectively), the other cells remained stable. At M12, T-cells CD8+ TEMRA (CD45RA+ CCR7−), and NK cells CD56+ CD3+ showed a significant increase, whereas NK cells CD56+ CD3− returned to baseline (*P* = 0.0124, *P* = 0.0356, and *P* = 0.2105, respectively).

### Evolution of Non-Anti-HLA Antibodies

Finally, we analyzed changes in the concentrations of other blood antibodies such as Ig, vaccine-induced antibodies (diphtheria, pneumococcus, and tetanus), and natural anti-ABO isohemagglutinins (Table 5). Ig decreased significantly during the follow-up and did not return to baseline levels at 12 months (*P* < 0.001). Regarding vaccine-induced antibodies, only patients with detectable antibody levels at enrollment (*n* = 11) were included in this analysis. At M3, only anti-diphtheria toxin IgG showed no significant change from baseline. At M12, almost all anti-pneumococcal serotypes 4, 6B, 9V,14, 18C, and 19F maintained their significant decrease (*P* = 0.0029, *P* = 0.0010, *P* = 0.033, *P* = 0.026, *P* = 0.045, and *P* = 0.012, respectively). For natural isohemagglutinins, anti-A IgG and anti-B IgM decreased significantly at M3 (*P* = 0.022 and *P* = 0.041, respectively). Almost all natural isohemagglutinins returned to baseline levels at M6 and M12, except for anti-A IgG at M6 (*P* = 0.007). Regarding infectious complications, 1 COVID-19 case was reported.

**Table 5:** Patient antibody status at baseline and follow-up time-points

Variables	Baseline	M3	P-value (baseline-M3)	M6	P-value (baseline-M6)	M12	P-value (baseline-M12)
Igs,							
Patients, *n*	13	13		13		11	
Blood sample, *n* (%)	11 (84.6)	10 (71.4)		11 (78.6)		9 (64.3)	
Concentration, g/l, median (IQR)	10 (9–16)	5 (5–6)	<0.001	6 (5–8)	<0.001	10 (7–12)	<0.001
Vaccination antibodies							
Patients, *n*							
Blood sample, *n* %	11 (78.6)	11 (78.6)		11 (78.6)		6 (42.9)	
Anti-diphtheria toxin IgG, UI/L, median (IQR)	0.22 (0.15–1.3)	0.27 (0.12–0.31)	0.168		0.043	0.46 (0.17–0.84)	0.066
Anti-pneumococcal serotype							
Serotype 4, mg/l, median (IQR)	0.5 (0.42–1.4)	0.54 (0.17–0.95)	0.01	0.4 (0.2–1)	0.014	0.39 (0.23–0.6)	0.029
Serotype 6B, mg/l, median (IQR)	2.3 (0.99–3)	0.96 (0.69–1.3)	0.001	0.94 (0.71–1.4)	0.001	1.2 (0.83–1.8)	0.01
Serotype 9V, mg/l, median (IQR)	0.92 (0.3–1.5)	0.88 (0.53–1.7)	0.049	0.53 (0.22–1.2)	0.024	0.8 (0.18–0.86)	0.033
Serotype 14, mg/l, median (IQR)	3.6 (2–11)	2.6 (1.1–5.5)	0.032	2.1 (0.85–6.6)	0.03	2.7 (0.74–4.9)	0.026
Serotype 18C, mg/l, median (IQR)	1.3 (0.46–2.4)	0.58 (0.27–1.3)	0.017	0.73 (0.27–0.8)	0.001	0.53 (0.21–0.89)	0.045
Serotype 19F, mg/l, median (IQR)	2.7 (2.2–6.1)	1.1 (0.68–2.1)	0.007	0.97 (0.68–1.6)	0.005	1.45 (1–1.8)	0.012
Serotype 23F, mg/l, median (IQR)	1 (0.31–3.1)	0.59 (0.2–1.6)	0.026	0.47 (0.32–1.5)	0.021	0.88 (0.42–1.4)	0.155
Anti-tetanus toxin IgG, UI/l, median (IQR)	1.19 (0.15–1.72)	0.80 (0.65–1.04)	<0.001	0.68 (0.51–0.84)	<0.001	0.97 (0.65–1.25)	0.001
Isohemagglutinins antibodies							
Patients, *n*							
Blood sample, *n*, %							
Anti-A hemagglutinin							
Patients, *n*	10	10		10		8	
IgG, UI/l, median (IQR)	40.0 (5.57–115.44)	21.5 (4.04–60.32)	0.022	17.13 (5.43–33.63)	0.007	25.99 (4.42–131.56)	0.17
IgM, UI/l, median (IQR)	15.05 (11.95–18.32)	12.27 (12–13.03)	0.055	11.85 (10.55–14.76)	0.119	17.45 (16.14–38.03)	0.194
Anti-B hemagglutinin, UI/l, median (IQR)							
Patients, *n*	8	8		8		7	
IgG, UI/l, median (IQR)	15.80 (5.56–20.48)	12.64 (5.20–23.90)	0.331	13.87 (7.07–21.74)	0.169	16.72 (6.38–22.27)	0.078
IgM, UI/l, median (IQR)	18.79 (11.08–42.13)	12.66 (9.38–32.15)	0.041	10.05 (7.97–38.08)	0.052	20.90 (11.10–27.29)	0.07

IQR, interquartile range; M, month.

### Follow-Up

Five of the patients enrolled in our study underwent transplantation, 2 of whom were included in the first phase. The first patient was treated with 4 doses of 4 mg/kg and transplanted 36 months after the first infusion, the second was treated with 4 doses of 8 mg/kg and transplanted 23 months after the first infusion (Supplementary Table S2). The remaining 3 were from the second phase and were transplanted 11, 17, and 24 months after the first infusion of daratumumab. The 5 patients are currently alive with functional allografts.

## Discussion

We report herein the first study using daratumumab, a humanized CD38-specific monoclonal antibody, to desensitize patients awaiting kidney transplantation with cPRA > 95%. Our study showed a rapid and significant decrease in cPRA, the number of anti-HLA, and their MFI max 3 months after the first infusion, although almost all returned to baseline values at M6. On the other hand, less than half of the patients had a durable response at 6 months.

Our escalating dose strategy was primarily used to assess the safety of daratumumab in kidney transplant candidates. Infusion-related reactions were the most common AEs and occurred immediately after the first infusion, as previously observed in larger cohorts of patients with myeloma treated with daratumumab.^10^ We did not report any severe TEAEs, demonstrating the safety of daratumumab in patients awaiting kidney transplantation and confirming the results of previous reports in isolated patients awaiting kidney transplantation or recipients of kidney transplantation.^12^^,^^17^^,^^18^ A recent report by Mayer *et al.*^22^ on another anti-CD38 antibody, felzartamab, used for the treatment of ABMR after kidney transplantation showed similar first dose reactions.

In the second phase, the multiple myeloma induction protocol was replicated with 8 weekly infusions of 16 mg/kg daratumumab to test its ability to reduce anti-HLA antibody levels and improve access to transplantation.^10^ Three months after the first infusion, we observed a rapid and significant reduction in almost all anti-HLA markers except cPRA 2000. This improvement was transient because levels returned to baseline 6 months later and remained so through M12. Presensitized animals treated with daratumumab also showed a similar short-term reduction in DSA antibodies, which quickly rebounded to their baseline levels by M12.^12^ More recently, isatuximab, a murine chimeric anti-CD38 antibody, has been used to desensitize 23 patients who had cPRA > 99.9% and awaiting kidney transplantation in 2 distinct cohorts.^19^ No SAEs were reported and cPRA was reduced to target levels in 40% of treated patients. At the end of the study, 17% of the patients received a transplant. Similarly, Mayer *et al.*^22^ reported in their study a modest decrease in MFI values for peak DSA, total IgG, and IgM, but with no SAE.

Currently, prioritization strategies (kidney allocation system) do not optimize allograft access for kidney transplant candidates with cPRA > 99.9%. In our study, daratumumab induced a durable response, defined as a decrease of at least 1% in cPRA at each time point, in less than half of the patients at M6 and in one-third of the patients at M12. Nevertheless, a reduction in cPRA from 99.9% to 99.8% or 99.7% could increase the transplantation rate by 12% and 20%, respectively.^20^ Such a small reduction is likely to give patients a better chance, and even the transient improvements could be seen as a gateway to a life-saving transplantation in highly immunized recipients.^12^^,^^14^^,^^18^ Kidney transplantation planned 2 to 4 months after the first infusion of daratumumab should be discussed in patients with limited access to transplantation.

The rebound of anti-HLA antibodies following the early decrease observed after daratumumab infusion suggests a potential role for daratumumab in the humoral compensation described in patients on proteasome inhibitors, which causes increase in B and T follicular helper cells.^23^ In a previous work, we have shown that belatacept blocks CD28-mediated activation of T follicular helper cells in an autologous T follicular helper cell memory B-cell model, and reduces the percentage of blood effector B-cells and activated T follicular helper cell (PD1+ ICOS+) in renal allograft recipients.^24^ The combination of daratumumab with belatacept in sensitized patients awaiting kidney transplantation may therefore stabilize anti-HLA antibody levels after an initial decline by reducing humoral compensation (ongoing work NCT05145296).^25^^,^^26^

Daratumumab has also been shown to be useful in refractory autoimmune diseases, systemic lupus, autoimmune cytopenia, MDA5-positive dermatomyositis, ANCA-associated vasculitis, Sjögren's syndrome, uncontrolled steroid-dependent nephrotic syndrome, and refractory posttransplant nephrotic syndrome associated with autoantibody decrease.^11^^,^^13^^,^^27^, ^28^, ^29^ However, and despite its primary efficacy, disease relapse, such as nephrotic syndrome and autoimmune cytopenia, has been observed and is often associated with the persistence of CD38-negative B-cells that escape drug depletion of plasma cells. Therefore, alternative sequential therapeutic strategies could combine anti-B-cell, anti-CD20 and anti-CD38 antibodies to reduce or even prevent plasma cell synthesis of anti-HLA antibodies.^13^^,^^29^^,^^30^

The risk of infection complicating daratumumab treatment in patients with multiple myeloma remained comparable to other treatments with stable polyclonal IgG levels, despite the reduction in normal plasma cells.^31^^,^^32^ In contrast, daratumumab caused hypogammaglobulinemia in patients with autoimmune diseases.^29^^,^^30^ In our patients, normal plasma cell levels decreased significantly at M1 and returned to baseline levels at M3. At the same time, we observed a significant decrease in Ig and vaccine-induced antibodies, which slowly returned to baseline levels at M12 without any infectious episodes. This result must be taken with caution because low Ig concentrations are likely to increase the rate of infection, the second leading cause of mortality in dialysis patients.^33^, ^34^, ^35^

We also evaluated the effect of daratumumab on anti-A/B isoagglutinins. To date, daratumumab has been considered the most effective therapy for peripheral red cell aplasia after ABO-mismatched bone marrow transplantation due to persistent antidonor hemagglutinins, with daratumumab significantly reducing these antibody levels.^36^^,^^37^ Such a reduction in hemagglutinins by daratumumab has also been reported after ABMR following ABO-incompatible kidney transplantation.^38^^,^^39^ We showed here that almost all hemagglutinins decreased within 3 months after daratumumab treatment. The combination of daratumumab with apheresis as a rescue therapy in ABO-incompatible kidney transplantation to lower initially high hemagglutinin levels may be a valid approach when transplantation is abandoned. The use of felzartamab should also be discussed for the treatment of antibody-mediated rejection after ABO-mismatched transplantation.^22^

Phenotypic analysis of PBMCs demonstrated that daratumumab induced a rapid reduction of cells with the highest CD38 surface expression, particularly plasma cells and NK cells. The reduction in plasma cells was transient, suggesting their susceptibility to daratumumab-mediated lysis and that circulating plasma cells are more sensitive to daratumumab than bone marrow plasma cells, which in turn is proportional to their CD38 expression level.^32^ As in multiple myeloma, patients with systemic lupus, and healthy donors, NK cells decreased for 6 months, suggesting their sensitivity to daratumumab-mediated lysis, because CD38 is expressed in up to 70% of NK cells where it physically or functionally cluster with FcγRIII/CD16, a critical membrane molecule.^27^^,^^40^ A comparable effect on NK cells was also evidenced in the recent report on anti-CD38 antibody for treatment of postrenal transplantation ABMR.^22^ Interestingly, NK cell functionality did not appear to be affected by daratumumab treatment in multiple myeloma patients.^40^ We also confirmed the absence of T-cell alterations highlighted in preclinical animal models, but showed a rapid decrease in CD8+ TEMRA, which expressed CD38+ in up to 75% of cells before treatment (data not shown), and in T-reg, both involved in humoral and cellular rejection after transplantation.^41^^,^^42^ Isatuximab therapy reduced the number of CD38+ plasmablasts, plasma cells, and NK cells and significantly reduced the number of HLA-specific IgG-producing memory B-cells; however, only 40% of patients had their cPRA reduced to target levels.^19^

We did not routinely dilute sera of hyperimmunized patients as suggested in the US.^43^ We are aware that MFI is not a perfect test for the assessment of anti-HLA antibodies and that dilution may facilitate the interpretation of anti-HLA concentration in such patients and reduce the risk of variations caused by the differences in batches.^43^ However, all our tests were performed centrally in 1 laboratory where the coefficients of variation were specifically studied. As for MFI of 500 to 1500, the variations could reach around 30%, but for MFI of more than 1500, the observed variations were around 10% (personal data). In addition, all sera were pretreated with EDTA to eliminate the prozone effect.

In summary, this clinical trial was designed to evaluate the safety and benefit of the anti-CD38 monoclonal antibody, daratumumab in the desensitization of highly immunized patients awaiting kidney transplantation. We demonstrated a rapid and significant, albeit transient, reduction in anti-HLA antibodies and other antibodies, including Ig, vaccine-induced antibodies, and natural isohemagglutinins, with less than 40% of the response being sustained. The phenotypic changes in PBMC correlated with the significant and sustained reduction in anti-HLA antibodies. The small effect of daratumumab on pretransplant HLA antibodies with no effect on the memory response (rebound); and the potential secondary effects on infectious complications due to its nonselective effect on the long-lived HLA antibodies producing plasma cells may seriously limit the use of daratumumab before transplantation. These findings need to be further investigated to select the best responders and subsequently tailor their treatment and the actual use of daratumumab alone in desensitization.

## Disclosure

All the authors declared no competing interests.

## Supplementary Material

Supplementary File (PDF) **Figure S1.** Gating strategy. **Figure S2.** Evolution of anti-HLA characteristics between baseline and M3 after the first infusion, each curve represents a patient. **Figure S3.** Evolution of anti-HLA characteristics between baseline and M6 after the first infusion, each curve represents a patient. **Table S1.** Anti-HLA characteristics according to intent-to-treat and per-protocol analysis. **Table S2.** kidney allograft transplantation characteristics.

## Footnotes

Supplementary File (PDF)
**Figure S1.** Gating strategy. **Figure S2.** Evolution of anti-HLA characteristics between baseline and M3 after the first infusion, each curve represents a patient. **Figure S3.** Evolution of anti-HLA characteristics between baseline and M6 after the first infusion, each curve represents a patient. **Table S1.** Anti-HLA characteristics according to intent-to-treat and per-protocol analysis. **Table S2.** kidney allograft transplantation characteristics.
