# Supplementary material for: Open-Label Phase 1/2 Study of Daratumumab-Based Desensitization Before Kidney Transplantation
Source: Kidney Int Rep. 2024 Aug 26;9(11):3250–64. doi: 10.1016/j.ekir.2024.08.020 (PMC11551132; doi:10.1016/j.ekir.2024.08.020)
Supplement: Supplementary File (PDF) — Figure S1. Gating strategy. Figure S2. Evolution of anti-HLA characteristics between baseline and M3 after the first infusion, each curve represents a patient. Figure S3. Evolution of anti-HLA characteristics between baseline and M6 after the first infusion, each curve represents a patient. Table S1. Anti-HLA characteristics according to intent-to-treat and per-protocol analysis. Table S2. kidney allograft transplantation characteristics. [file mmc1.pdf]

Supplementary material

1- Supplementary tables

- Supplementary data Table S.1: Anti-HLA characteristics according to intent-to-treat and per-protocol analysis

	Baseline	Month 1	P-value (baseline-M1)	Month 3	P-value (baseline-M3)	Month 6	P-value (baseline-M6)	Month 12	P-value (baseline-M12)
Intent-to-treat analysis									
Analysis with multiple imputation									
**Patients, N**	14	14		14		14		14	
Blood sample, N, %	14 (100)	14 (100)		14 (100)		14 (100)		14 (100)	
**cPRA**									
2000 threshold, %, median [IQR]	98 [85-100]	97 [82-99]	0.674	97 [86-99]	0.364	96 [90-99]	0.67	98 [92-100]	0.357
10000 threshold, %, median [IQR]	65 [56-84]	57 [21-83]	0.126	57 [23-79]	0.027	57 [27-84]	0.175	61 [43-85]	0.895
**MFI sum**									
class I and class II, median [IQR]	296654 [183796-459407]	302589 [132763-379182]	0.016	276527 [128888-317537]	0.001	280008 [138277-314512]	0.032	307512 [145597-377266]	0.358
class I, median [IQR]	224413 [125264-388411]	173032 [73519-316940]	0.019	133389 [64600-243921]	0.001	156493 [75296-267207]	0.012	204213 [101242-283842]	0.443
class II, median [IQR]	71058 [39830-131834]	61905 [29536-122022]	0.081	50826 [36149-117749]	0.028	50176 [41963-119766]	0.302	53273 [44024-108133]	0.367
**MFI max**									
class I and class II, median [IQR]	20811 [19799-24014]	22287 [15622-23925]	0.362	20933 [14545-23368]	0.07	20879 [17602-23016]	0.197	21337 [19140-22954]	0.651
class I, median [IQR]	19399 [10855-22267]	16733 [9857-21138]	0.155	16648 [9413-21392]	0.01	13686 [12038-20840]	0.04	16274 [11501-21053]	0.089
class II, median [IQR]	20126 [10485-23116]	16279 [7216-23654]	0.687	17180 [8669-23277]	0.501	20524 [10302-21867]	0.749	20841 [7681-22788]	0.881
**Anti HLA number**									
class I and class II, median [IQR]	61 [45-83]	50 [35-70]	<0.001	52 [36-66]	<0.001	54 [34-69]	0.014	57 [47-73]	0.332
class I, median [IQR]	43 [32-56]	37 [19-53]	<0.001	36 [21-52]	<0.001	37 [20-53]	0.007	39 [27-58]	0.558
class II, median [IQR]	17 [6-29]	13 [8-19]	0.006	15 [5-21]	0.008	15 [12-24]	0.471	16 [12-23]	0.352
Per-protocol analysis									
**Patients, N**	11	11		11		11		11	
Blood sample, N, %	11 (100)	11 (100)		11 (100)		11 (100)		11 (100)	
**cPRA**									
2000 threshold, %, median [IQR]	99 [95-100]	99 [92-100]	0.904	97 [88-100]	0.314	98 [93-100]	0.6	99 [93-100]	0.197
10000 threshold, %, median [IQR]	80 [57-98]	73 [56-98]	0.151	71 [48-94]	0.002	80 [38-94]	<0.001	79 [56-94]	0.281
**MFI sum**									
class I and class II, median [IQR]	410941 [217711-559005]	319457 [194266-415144]	0.008	276980 [146348-462118]	<0.001	281352 [160798-538071]	0.008	318340 [168918-579956]	0.212
class I, median [IQR]	247617 [148482-452791]	267535 [78688-339011]	0.01	226409 [67540-366650]	<0.001	180681 [80514-418305]	0.006	238425 [127409-471823]	0.25
class II, median [IQR]	70996 [46681-145003]	62242 [34621-127185]	0.127	51421 [41266-125243]	0.029	48470 [45655-132444]	0.237	52793 [44828-147020]	0.367
**MFI max**									
class I and class II, median [IQR]	22276 [20240-24152]	22869 [19795-24998]	0.988	22387 [19834-23613]	0.129	21102 [20657-23040]	0.056	22041 [20647-23414]	0.665
class I, median [IQR]	20240 [18398-22268]	19795 [14264-23536]	0.222	19834 [14085-22036]	0.012	19475 [12460-21817]	0.024	19162 [15586-21092]	0.041
class II, median [IQR]	21717 [14082-23940]	22532 [12407-24998]	0.642	21881 [8918-23613]	0.816	20887 [10302-22996]	0.829	22041 [8162-23414]	0.622
**Anti HLA number**									
class I and class II, median [IQR]	61 [45-84]	50 [35-71]	<0.001	45 [36-67]	<0.001	50 [34-67]	0.002	57 [48-72]	0.236
class I, median [IQR]	44 [32-56]	37 [19-53]	<0.001	37 [21-52]	<0.001	37 [20-53]	0.002	39 [27-58]	0.369
class II, median [IQR]	17 [6-29]	14 [11-21]	0.039	15 [4-21]	0.008	14 [12-22]	0.255	16 [14-25]	0.355

- Supplemental data Table S2 : kidney allograft transplantation characteristics

**Transplantation**	**#1**	**#2**	**#3**	**#4**	**#5**
**Desensitisation**					
Protocol step	I	I	II	II	II
Daratumumab dose	4 mg/kg	8 mg/kg	16 mg/kg	16 mg/kg	16 mg/kg
**Donor**					
Deceased	yes	yes	yes	yes	yes
Age, years	76	49	76	64	67
HLA mismatch, N	4	5	6	1	8
**At the time of transplantation**					
Delay from inclusion, months	36	23	24	17	11
Anti-HLA donor specific antibodies	no	yes	yes	no	yes
Number	-	3	3	-	1
MFI max	-	2700	2140	-	7800
Crossmatch, lymphocytoxicity					
T crossmatch	negative	negative	negative	negative	negative
B crossmatch	negative	negative	negative	negative	negative
Immunosuppressive treatment					
Induction	yes	yes	yes	yes	yes
Thymoglobulin	yes	yes	yes	yes	yes
Intravenous immunoglobulins	no	yes	yes	no	yes
Rituximab	no	yes	yes	no	yes
Plasmapheresis	no	yes	yes	no	yes
Maintenance					
Calcineurin inhibitors	tacrolimus	tacrolimus	tacrolimus	tacrolimus	tacrolimus
Mycophenolate mofetil	yes	yes	yes	yes	yes
Steroids	yes	yes	yes	yes	yes
**Follow-up**					
Acute rejection	yes	no	yes	no	yes
Delay from transplant	6	-	5	-	4
Type of rejection	mixed	-	ABMR*	-	ABMR*
End of follow-up - April 2024					
Delay from transplant, months	13	23	20	12	31
Death	no	no	no	no	no
eGFR** , ml/min/1.73m2 (CKD-epi)	31	58	19	92	66

* acute antibody mediated rejection

** estimated glomerular filtration rate

2- **Supplementary figures**

Figure S.1 : gating strategy

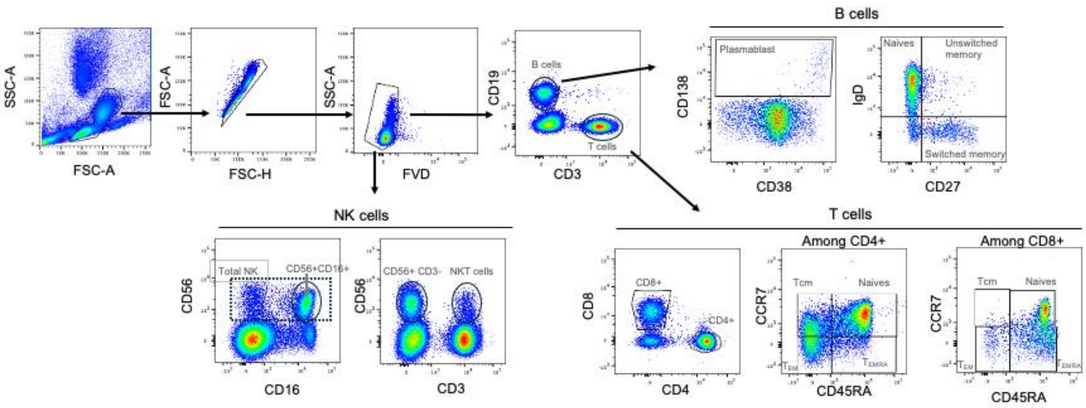

Legend to Figure S.1: Gating strategy - Lymphocytes were first gated on low-side scatter and forward scatter and then single cells were selected. Live cells were selected by low expression of fixable viability dye (FVD). Total T cells were defined by CD3+ expression and then divided into CD4 and CD8 populations. Within these two T cell populations, the markers CD45RA and CCR7 identified four populations: central memory (Tcm CCR7+CD45RA-), effector memory (TEM CCR7-CD45RA-), naive (CCR7+CD45RA+) and terminally differentiated effector memory (TEMRA CCR7-CD45RA+). Total B cells were defined as CD19+ lymphocytes. The following CD19+ B cell subsets were identified: naïve B cells as CD27-IgD+, switched memory B cells as CD27+IgD-, unswitched memory B cells as CD27+IgD+ and plasmablasts as CD138+. Total NK cells were defined as CD56+. NK subtype CD56+CD16-, CD56+CD3- and NKT cells identified as CD56+CD3+ were gated among all PBMC.

Figure S.2: Anti HLA three months evolution

S.2.A : cPRA 2000

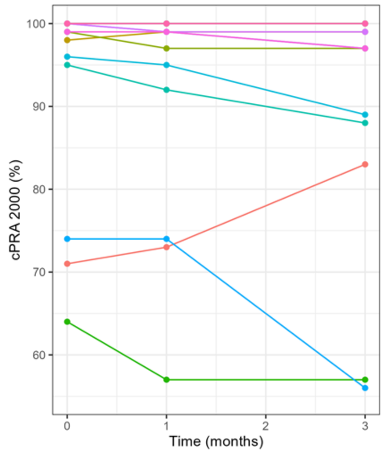

S.2.B : cPRA 10000

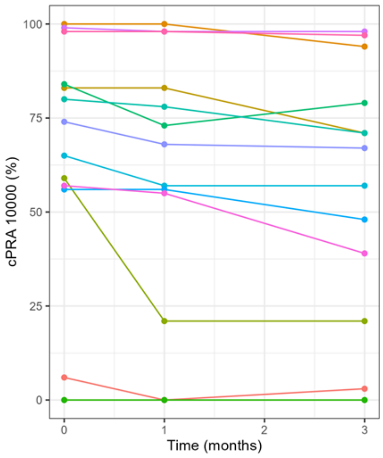

S.2.C : number of anti-HLA

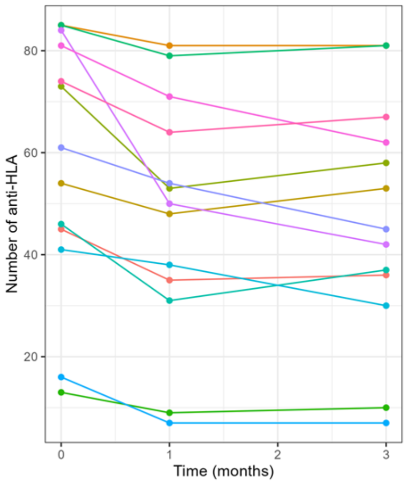

S.2.D : MFI max

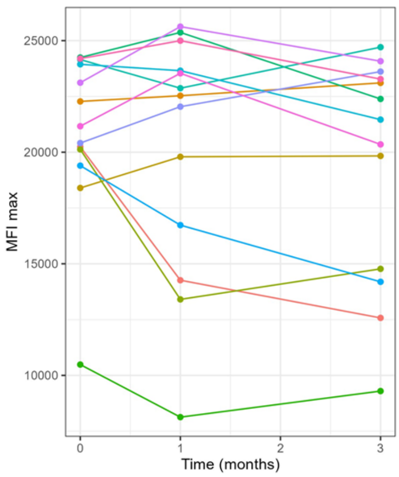

S.2.E : MFI sum

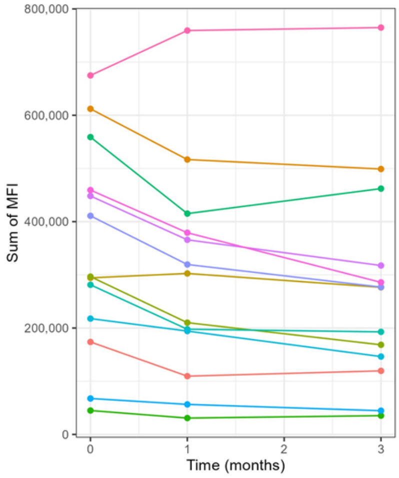

Legend to figure S.2: Evolution of anti-HLA characteristics between baseline and three months after the first infusion, each curve represents one patient.

S.1.A: cPRA 2000 remained stable (P=0.185); S.1.B: cPRA 10000 decreased significantly (P=0.003); S.1.C: total number of anti-HLA antibodies decreased significantly (P<0.001); S.1.D: total MFI max decreased significantly (P=0.05); S.1.E: total MFI sum decreased significantly (P<0.001).

Figure S.3 : Anti HLA six months evolution

S.3.A : cPRA 2000

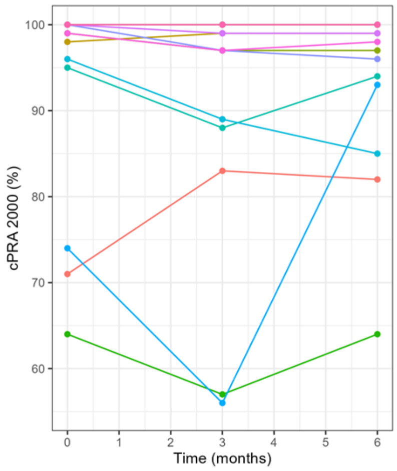

S.3.B : cPRA 10000

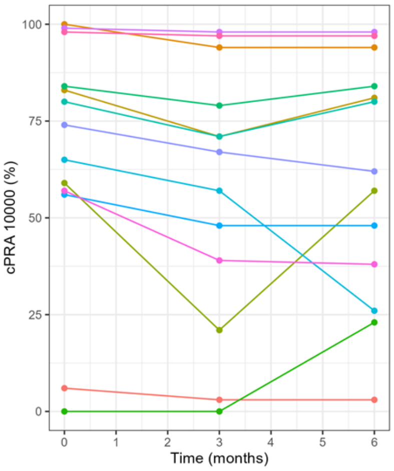

S.3.C : number of anti-HLA

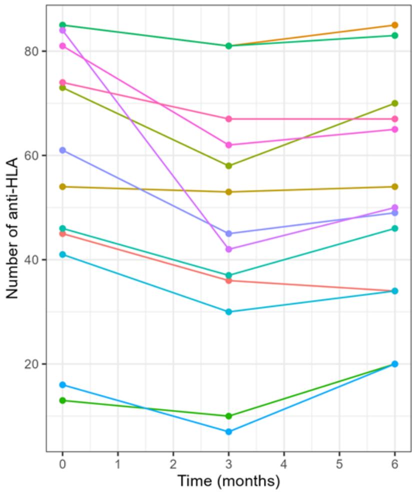

S.3.D : MFI max

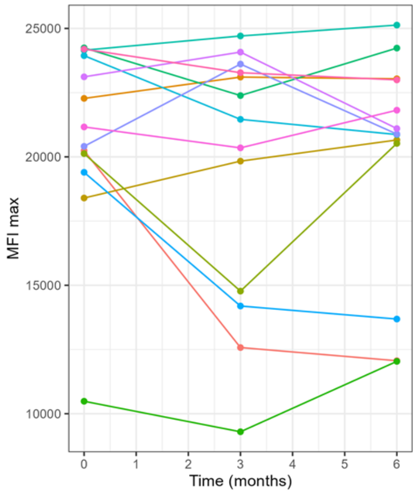

S.3.E : MFI sum

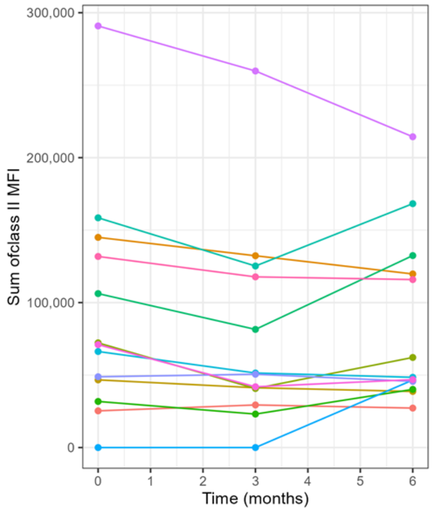

Legend to figure S.3: Evolution of anti-HLA characteristics between baseline and six months after the first infusion, each curve represents one patient.

S.2.A: cPRA 2000 remained stable (P=0.668); S.2.B: cPRA 10000 returned to baseline (P=0.074); S.2.C: total number of anti-HLA antibodies decreased significantly (P=0.007); S.2.D: total MFI max returned to baseline (P=0.17); S.2.E: MFI sum decreased significantly (P=0.005).
